# Telemedicine: Inter-Method Agreement Between In-Person Consultations and Video Recordings When Diagnosing Benign Paroxysmal Positional Vertigo

**DOI:** 10.3390/jcm14072495

**Published:** 2025-04-06

**Authors:** Ali A. Melliti, Rajneesh Bhandari, Anita Bhandari, Mustafa Karabulut, Ellen Rikers, Sophie Paredis, Sophie Vanbelle, Raymond van de Berg

**Affiliations:** 1 Division of Vestibular Disorders, Department of Otorhinolaryngology and Head and Neck Surgery, School for Mental Health and Neuroscience, Maastricht University Medical Center, 6229 HX Maastricht, The Netherlands; mustafa.karabulut@mumc.nl (M.K.);; 2 NeuroEquilibrium Diagnostic Systems Pvt Ltd., Jaipur 302018, India; 3 Vertigo and Ear Clinic, Jaipur 302018, India; 4 Department of Methodology and Statistics, CAPHRI, Maastricht University, P. Debyeplein, 1, Maastricht, 6229 HA, The Netherlands

**Keywords:** telemedicine, benign paroxysmal positional vertigo, nystagmus

## Abstract

**Objective:** To investigate the inter-method agreement between in-person consultations and video recordings when diagnosing BPPV. **Methods:** Two experienced vestibular clinicians (clinician A and B) evaluated patients for the presence and type of BPPV, using the TRV chair (Interacoustics, Middlefart, DK), at a tertiary referral center. During these in-person consultations, diagnostic maneuvers and eye movements were recorded, and a diagnosis was made. Both clinicians independently evaluated their cases again, during two video review sessions (Video Review 1 and Video Review 2). These sessions were conducted one month apart. Both clinicians were blinded to patient information and medical history during the analysis and did not have access to sound recordings. They were asked to provide a new diagnosis, based on the videos alone. Inter-method and intra-observer agreement for BPPV diagnoses between in-person consultations and video reviews were assessed using the percentage of agreement and Cohen’s kappa. An independent analysis of all patients’ eye movements was conducted to identify patterns that might have influenced agreement between in-person consultation diagnoses and the two video reviews by clinicians. **Results:** During the in-person consultations, each clinician evaluated 100 patients. Clinician A diagnosed BPPV in 40% of the cases, while clinician B diagnosed it in 19% of the cases. Considering the inter-method agreement, clinician A agreed on 81% (95% CI (73, 89)) and 77% (95% CI (69, 85)) of the cases with associated kappa coefficients of 0.67 (95% CI (0.55, 0.79)) and 0.63 (95% CI (0.51, 0.75)) between in-person consultations and Video Reviews 1 and 2, respectively. For clinician B, the percentages of agreement were, respectively, 86% (95% CI (79, 93)) and 84% (95% CI (77, 91)), with corresponding kappa coefficients of 0.55 (95% CI (0.36, 0.74)) and 0.51 (95% CI (0.32, 0.70)). As for the intra-observer agreement, clinician A achieved an intra-observer agreement of 84% (95% CI (77, 91)) with kappa = 0.74 (95% CI (0.63, 0.85)), while clinician B achieved a slightly higher intra-observer agreement of 90% (95% CI (84, 96)) with kappa = 0.67 (95% CI (0.51, 0.83)). Descriptive analysis of the eye movement revealed that both clinicians showed high diagnostic consistency for “no BPPV” in cases without provoked nystagmus (78/86, 91%) even when spontaneous nystagmus was present, and for posterior canal BPPV (37/78, 47%) when characteristic nystagmus was observed. However, disagreement was noted for horizontal canal BPPV (15 cases) and in scenarios with subjective BPPV (2 cases) or purely vertical nystagmus (11/31 cases, 35%). **Conclusions:** This study showed the feasibility of using video recordings when diagnosing BPPV. It demonstrates that BPPV might be reliably diagnosed in a telemedicine setting. However, careful consideration must be given to certain factors during the protocol’s design to improve the diagnostic process.

## 1. Introduction

Benign paroxysmal positional vertigo, also known as BPPV, is an inner ear-related vestibular disorder that represents 20–30% of diagnoses in a specialized vestibular clinic [1,2]. It is believed to be caused by the detachment and displacement of otoconia from the utricle to the semicircular canal(s) [3]. BPPV provokes a transient spinning sensation triggered by changes in head position [4,5]. The diagnostic criteria for BPPV require a history of vertigo or dizziness and provoked positional nystagmus during positional maneuvers [3,5]. Due to the nature of BPPV, it can be treated effectively with canal repositioning maneuvers at any specialized outpatient clinic. This includes, e.g., the Epley, Semont, and Semont-Plus maneuver for posterior canal BPPV [6,7,8,9] and the Gufoni, Appiani, and Barbecue maneuvers for horizontal canal BPPV [10,11]. Despite the high success rate of the treatment, BPPV is tied with significant health care costs [5]. These high costs can be explained by the delayed diagnosis, unnecessary diagnostic testing, and treatment procedures [5,12,13].

Telemedicine is one solution that has been introduced to overcome these challenges [14]. Its use has significantly expanded in clinical settings, especially during the COVID-19 pandemic [15,16,17,18]. Hence, assessing the reliability and validity of this technique is crucial to ensure that it is comparable to traditional clinical practices. Evaluating patients with dizziness, for example, often requires a thorough investigation of their medical history, physical examination, laboratory tests (e.g., videonystagmography), and other ancillary tests such as imaging [19,20]. Consequently, conducting assessments online may compromise the quality of care. After all, depending on the setting, additional variables are introduced. For example, a patient can be assessed by a (non-expert) clinician during an in-person consultation, but the recordings of this assessment can be evaluated remotely by another (expert) clinician. This results in additional variables related to the methods (in-person consultation and recordings) and the clinicians (non-expert and expert), compared to a “traditional” consultation. As a consequence, the inter-method variability (in-person consultation versus recordings) and inter-observer variability (non-expert versus expert) might influence the diagnostic process. Previously, Shah et al. (2019) investigated the inter-method agreement. It was shown that smartphone-based video recordings of patients’ eye movements exhibit high sensitivity and specificity in diagnosing BPPV [14]. However, the study had a relatively small sample size.

Therefore, this study aimed to evaluate the inter-method agreement between in-person consultations and video recordings when evaluating patients for BPPV using a larger sample size. To accurately quantify the inter-method agreement, the inter-observer variability was eliminated: the same examiner performed the in-person consultations and assessment of the video recordings.

## 2. Materials and Methods

### 2.1. Study Design

**Figure 1:**
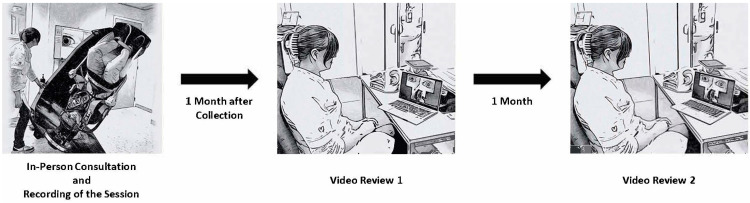
Overview of study design.

Two experienced vestibular clinicians evaluated patients for the presence and type of BPPV at a tertiary referral center at Maastricht University Medical Center, the Netherlands. Initially, every patient underwent an in-person consultation, during which the Dix–Hallpike maneuver and supine roll test were performed, and a diagnosis was made. Video recordings were made of all consultations. After patient inclusion, each clinician conducted two separate video reviews of their own patients, with a one-month interval between them. During both reviews, they provided a diagnosis for each patient while blinded to their previous assessments (Figure 1). Diagnoses from the three time points (in-person consultation, Video Review 1, and Video Review 2) were compared.

### 2.2. Clinicians

Two vestibular laboratory technicians were selected based on years of practice in vestibular evaluation, specifically in diagnosing and treating BPPV. Each clinician had at least 20 years of experience. The types of patients examined by both clinicians differed because of the triage system of the hospital. Therefore, it was hypothesized that the percentage of BPPV patients might differ between the clinicians’ populations.

### 2.3. Patients

Two hundred patients were included in this study: 100 patients for each clinician. Patient inclusion criteria comprised individuals who were at least 18 years of age and underwent vestibular testing because of vestibular symptoms. Exclusion criteria were an inability to undergo the diagnostic maneuvers and insufficient quality of video recordings.

### 2.4. Testing Procedures

For standardization purposes, the TRV chair (Interacoustics, Middlefart, Denmark) was used to perform the diagnostic maneuvers, and Visualeyes video goggles (Interacoustics, Middlefart, Denmark) to record eye movements. In addition, a high-definition room camera (Logitech, Lausanne, Switzerland) was synchronized with the TRV system to record the patient’s head position during testing. The diagnostic protocol sequence comprised 5 examinations, all of which were recorded: (1) spontaneous nystagmus in an upright sitting position; (2) spontaneous nystagmus in a supine position; (3) supine roll test to the right and left sides; (4) Dix–Hallpike test to the right; and (5) Dix–Hallpike test to the left. Each clinician diagnosed the patients during the in-person consultation. The following parameters were collected: presence of BPPV, affected side(s), affected canal(s), and BPPV type (geotropic or apogeotropic, in the case of horizontal canal BPPV).

The videos were edited using Adobe Premiere Pro 2022 (Adobe, San Jose, CA, USA) to standardize recordings without compromising testing quality. Videos were muted to minimize potential bias introduction. After all, the clinicians were instructed to not mention the diagnosis while recording. However, conversations while recording (e.g., with patients) could sometimes still reveal (part of) the diagnosis. On average, all recordings lasted approximately 1 min, except for the supine roll, which lasted approximately 1 min and 30 s.

**Figure 2:**
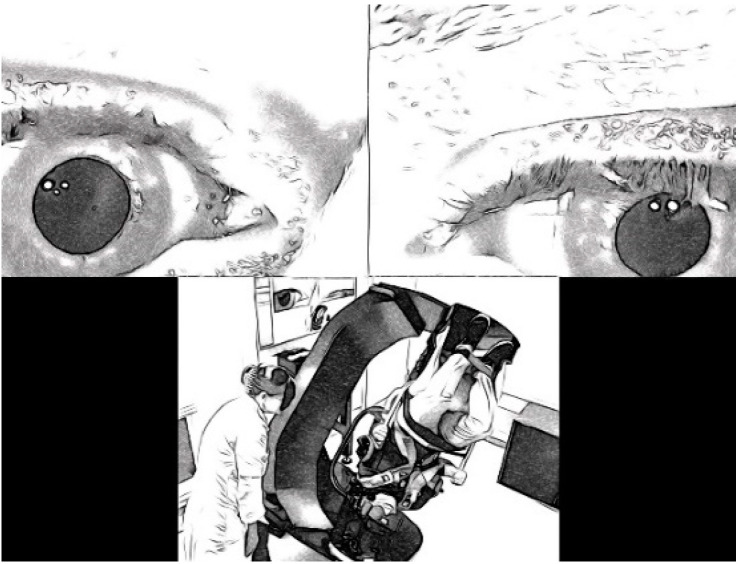
Clinician’s view during video reviews.

One month after examining their last patient, both clinicians independently reviewed their recorded cases (Video Review 1, see Figure 1) and provided a diagnosis again (Figure 2). Each patient’s videos were placed in an anonymized folder, with the sequence of videos not randomized to reflect the clinical routine procedure (i.e., always first examining spontaneous nystagmus in upright position). The order in which patients were reviewed was not randomized, but clinicians were blinded to patient information, medical history, and diagnoses. One month after Video Review 1, both clinicians repeated the procedure (Video Review 2). Again, clinicians provided a diagnosis for each patient. At the end of the video reviews, the clinicians were asked to fill out a reflection form. This comprised reflection on their experience evaluating BPPV, using only video recordings.

### 2.5. Descriptive Analysis of Eye Movements and Diagnostic Consistency

Two of the authors (AM and MK) analyzed the videos of all 200 cases. In consensus, the different eye movement patterns found during examination were described and categorized. Both authors were blinded from the clinicians’ diagnoses and had access to the sound recording of the patients. This consensus aimed to identify eye movement patterns that could have influenced the level of agreement between the in-person consultation diagnoses and the two video reviews of both clinicians.

### 2.6. Statistical Analysis

The results are summarized as mean and standard deviation for quantitative variables and proportions for qualitative variables. Inter-method agreement (i.e., in-person consultation vs. video reviews) and intra-observer agreement (i.e., Video Review 1 vs. Video Review 2) were reported. The agreements between two ratings were evaluated through the percentage of agreement and Cohen’s kappa coefficient for binary variables (e.g., presence of BPPV, yes/no) and nominal variables (a complete BPPV diagnosis). A complete BPPV diagnosis included an identification of (a) the presence of BPPV, (b) the affected canal(s), and c) the affected side(s) with BPPV. Confidence intervals were derived based on the delta method. The sample size was determined based on a presence of BPPV expected in 25% of the patients [1], a kappa coefficient of 0.90, and a 95% confidence interval width of 0.2 following the method described in Shoukri (2010) [21]. The kappa agreement levels were interpreted per Landis and Koch (1977): almost perfect (0.81–1.00), substantial (0.61–0.80), moderate (0.41–0.60), fair (0.21–0.40), slight (0–0.20), and poor < 0 [22]. Considering the benign nature of BPPV, the percentage agreement levels were categorized as follows: almost perfect (91–100%), good (81–90%), moderate (61–80%), fair (41–60%), and poor (0–40%).

A descriptive analysis summarized key observations from the consensus on eye movements and compared them to clinician diagnostic consistency. Analysis was made in R v4.1.2 (R Core Team, Vienna, Austria), SPSS v28.0.0.0 (IBM, Armonk, NY, USA), and Microsoft Excel 2016 (Microsoft, Redmond, WA, USA).

### 2.7. Ethical Approval

The ethical committee of Maastricht University Medical Center, the Netherlands (METC 2021-2938, approved on 27 December 2021) approved this study. Informed consent was obtained from all patients. This study was conducted in accordance with the legislation and ethical standards on human experimentation in the Netherlands and in accordance with the Declaration of Helsinki (amended version 2013).

## 3. Results

### 3.1. Patient Characteristics

Each clinician evaluated 100 patients in this study. During the in-person consultations, clinician A diagnosed BPPV in 40% of the cases, while clinician B diagnosed BPPV in 19% of the cases. The patient characteristics are shown in Table 1 and Table 2.

### 3.2. Agreement Between In-Person Consultations and Video Reviews

Clinician A demonstrated good agreement on the presence of BPPV in 88% (95% CI (82, 94)) and 90% (95% CI (84, 96)) between in-person consultations and Video Reviews 1 and 2, respectively (Table 3). Corresponding Cohen’s kappa coefficients were substantial: 0.75 (95% CI (0.62, 0.88)) and 0.80 (95% CI (0.68, 0.92)). Similarly, for clinician B, good percentages of agreement were observed: respectively, 89% (95% CI (83, 95)) and 87% (95% CI (80, 94)), with substantial and moderate Cohen’s kappa coefficients of 0.62 (95% CI (0.42, 0.82)) and 0.57 (95% CI (0.36, 0.78)).

When considering the complete BPPV diagnosis (presence of BPPV, with affected canal and side), clinician A demonstrated good and moderate agreement on 81% (95% CI (73, 89)) and 77% (95% CI (69, 85)) of the cases with associated substantial kappa coefficients of 0.67 (95% CI (0.55, 0.79)) and 0.63 (95% CI (0.51, 0.75)). For clinician B, the percentages of agreement were good: respectively 86% (95% CI (79, 93)) and 84% (95% CI (77, 91)), with corresponding moderate kappa coefficients of 0.55 (95% CI (0.36, 0.74)) and 0.51 (95% CI (0.32, 0.70)).

### 3.3. Intra-Observer Agreement

Table 3 further summarizes the intra-observer agreement regarding the presence of BPPV and the complete BPPV diagnosis. Clinician A achieved a good intra-observer agreement of 84% (95% CI (77, 91)) with a substantial kappa = 0.74 (95% CI (0.63, 0.85)) for a complete diagnosis, while clinician B achieved a slightly higher intra-observer agreement of 90% (95% CI (84, 96)) with a substantial kappa = 0.67 (95% CI (0.51, 0.83)).

### 3.4. Descriptive Analysis of Eye Movement Patterns

Table 4 provides a summary of key observations following the descriptive analysis of the obtained eye movements. First of all, it was found that both clinicians diagnosed “no BPPV” in 78/86 (91%) of cases where no nystagmus was provoked by diagnostic maneuvers, even in the presence of spontaneous nystagmus. In fact, 37/78 of these cases (47%) had spontaneous nystagmus in sitting or supine position.

It was also shown that both clinicians consistently diagnosed posterior canal BPPV in 37/48 (77%) of cases (left and right Dix–Hallpike analyzed separately) when the characteristic vertical upbeat nystagmus with torsional component toward the tested side was present. The 11 remaining cases demonstrated low or moderate nystagmus velocity and/or short nystagmus duration (less than three beats).

Furthermore, an inconsistent diagnosis of horizontal canal BPPV was often made (15 cases) in two types of scenarios: (1) the presence of nystagmus with a low or moderate velocity; (2) the presence of almost equal velocities obtained during supine rolls to the left and right. Finally, diagnostic consistency was also affected in cases with subjective BPPV (2/2 cases) and the presence of purely vertical nystagmus (11/31 cases, 35%).

### 3.5. Reflection on the Video Reviews

Clinician A indicated difficulties visualizing torsional components of the nystagmus through infrared recordings. In addition, clinician A found BPPV cases with slower nystagmus particularly challenging to assess without patient feedback about the experienced dizziness during maneuvers.

Clinician B noted that the large sample size might have added an additional burden, making it difficult to accurately evaluate all patients because of fatigue. Additionally, clinician B found some recordings of suboptimal quality due to artefacts such as blinking or goggle slippage.

## 4. Discussion

This study demonstrated an overall good level of agreement in diagnosing BPPV during in-person consultations and video reviews despite the absence of additional clinical information (i.e., case history and audio feedback).

Although the percentages of agreement between clinicians A and B were similar, clinician A exhibited higher Cohen’s kappa coefficient values than clinician B. This difference could be attributed to the different prevalence rates of BPPV [24]. As stated above, these prevalence rates were influenced by the triage system of the hospital. Due to the lower number of patients diagnosed with BPPV by clinician B, the kappa value became more sensitive to disagreements. Since this study was about the feasibility of telemedicine, it was preferred to use the percentage of agreement as the main outcome measure

The percentage of intra-observer and inter-method agreement were both good. This could imply that using telemedicine as a technique is feasible. Nevertheless, based on the descriptive analysis of the obtained eye movements, several considerations remain to be addressed to improve the diagnostic process of telemedicine (Table 5):

First, spontaneous nystagmus should be recorded in both sitting and supine position. After all, it is commonly observed in healthy patients [25]. This enables clinicians to account for the nystagmus during diagnostic maneuvers. This might provide important context and could help to avoid misdiagnoses. Second, a presence of crescendo–decrescendo vertical upbeat nystagmus with torsional component was found to be the most reliable indicator for consistently diagnosing posterior canal BPPV [3,5]. However, this characteristic nystagmus could be missed if it is subtle (e.g., low nystagmus velocity). Therefore, incorporating tracings of the nystagmus (horizontal, vertical, and torsional components) would provide additional objective measures to support the diagnosis [26]. In addition, objective measures of slow-phase velocity (SPV) can also assist clinicians in identifying the affected side in horizontal canal BPPV. Third, additional diagnostic tests, including the bow and lean test, may be needed to improve the diagnosis of horizontal canal BPPV [27,28,29,30]. Fourth, the diagnosis of subjective BPPV inherently requires access to the patient’s feedback during the maneuvers (e.g., reports of nausea or dizziness) [31,32]. One clinician noted that the lack of patient feedback complicated the evaluation of patients with subtle nystagmus. Therefore, this highlights the importance of incorporating patient feedback into the telemedicine protocol.

Finally, the diagnostic inconsistency in cases demonstrating purely vertical nystagmus was to be expected. This type of nystagmus can contribute to diagnostic uncertainty [33,34,35]. After all, vertical positional nystagmus can also be a sign of vestibular migraine or other central vestibular disorders [36,37,38]. The nystagmus characteristics of these disorders differ from BPPV regarding several parameters. Generally, in central vestibular disorders, the positional nystagmus is persistent (not crescendo–decrescendo) and purely vertical, horizontal, or torsional (not mixed vertical–torsional or horizontal–torsional). Furthermore, the velocity is often low [37,39]. In this study, the clinicians were trained to recognize these patterns. However, in clinic, the nystagmus pattern is not always perfectly clear in patients (e.g., in the case of low nystagmus velocity). In such cases, performing a repositioning maneuver may help confirm or reject the diagnosis [3].

Taking the results of this study into account, a BPPV telemedicine protocol enables BPPV identification similar to in-person consultation in the vast majority of cases. This approach could therefore be used to improve patient care, especially in areas with limited access to specialists in vestibular medicine [40].

### Limitations

Three limitations were identified in this study: Firstly, the cases were evaluated in order of collection, which could possibly introduce bias (e.g., patient body figure, clothes). However, due to the large sample size and time intervals between reviews, memory recall bias was likely minimal. Secondly, despite the good inter-method and intra-observer agreement, this large sample size may have placed a significant burden on the clinicians, which could have potentially affected diagnostic accuracy. Lastly, limited information available to clinicians may also have contributed to variability in their responses, potentially affecting the generalizability of the findings to real-world telemedicine applications. Therefore, it might be assumed that this study underestimated the agreement. Providing more information to the clinicians could increase agreement and improve diagnostic accuracy.

## 5. Conclusions

This study showed the feasibility of using video recordings when diagnosing BPPV. It demonstrates that BPPV might be reliably diagnosed in a telemedicine setting. However, careful consideration must be given to certain factors during the protocol’s design to improve the diagnostic process.

## Figures and Tables

**Table 1:** Patient characteristics collected by each clinician during in-person consultations.

Characteristics	Clinician A (*N* = 100)	Clinician B (*N* = 100)
Gender		
Female	67%	57%
Male	33%	43%
Age (Mean +/− SD)	57.3 +/− 14 years	58.5 +/− 13.9 years
Presence of BPPV	40%	19%

**Table 2:** BPPV diagnosis prevalence collected by each clinician during in-person consultations.

Characteristics	Clinician A (*N* = 40)	Clinician B (*N* = 19)
Clinical Presentation		
Subjective BPPV	2.5%	5.3%
Objective BPPV	97.5%	94.7%
Type of BPPV		
Posterior canal BPPV	60%	73.7%
Geotropic horizontal canal BPPV	2.5%	10.5%
Apogeotropic horizontal canal BPPV	10%	0%
Anterior canal BPPV	2.5%	5.3%
Multi-canal BPPV *	25%	10.5%

* Multi-canal BPPV included any combination of BPPV in different canals on the same or on the opposite side [23].

**Table 3:** Inter-method and intra-observer agreement analysis.

		Clinician A		Clinician B	
Agreement		Percentage of Agreement (%), 95% CI	Kappa, 95% CI	Percentage of Agreement (%), 95% CI	Kappa, 95% CI
In-person consultation vs. Video Review 1 (inter-method agreement)	Presence of BPPV	88, (82, 94)	0.75, (0.62, 0.88)	89, (83, 95)	0.62, (0.42, 0.82)
	Complete BPPV diagnosis *	81, (73, 89)	0.67, (0.55, 0.79)	86, (79, 93)	0.55, (0.36, 0.74)
In-person consultation vs. Video Review 2 (inter-method agreement)	Presence of BPPV	90, (84, 96)	0.80, (0.68, 0.92)	87, (80, 94)	0.57, (0.36, 0.78)
	Complete BPPV diagnosis *	77, (69, 85)	0.63, (0.51, 0.75)	84, (77, 91)	0.51, (0.32, 0.70)
Video Review 1 vs. Video Review 2 (intra-observer agreement)	Presence of BPPV	90, (84, 96)	0.79, (0.67, 0.91)	94, (89, 99)	0.79, (0.63, 0.95)
	Complete BPPV diagnosis *	84, (77, 91)	0.74, (0.63, 0.85)	90, (84, 96)	0.67, (0.51, 0.83)

*: “complete BPPV diagnosis” included an identification of the presence of BPPV, including canal(s) and side(s) affected by BPPV.

**Table 4:** Key observations following descriptive analysis of obtained eye movements.

Key Observation	Additional Remarks
The absence of nystagmus provoked by a diagnostic maneuver (*N* = 86) was often consistent with the absence of a BPPV diagnosis (*N* = 78).	In 37/78 (47.43%) cases, a spontaneous nystagmus was present.
The presence of crescendo–decrescendo vertical upbeat nystagmus with torsional component toward the tested side (*N* = 48) often led to a consistent BPPV diagnosis (*N* = 37) *.	In the inconsistent cases (11/48, 22.91%), a low/moderate nystagmus velocity and/or short nystagmus duration (less than 3 beats) was present *.
The presence of low/moderate nystagmus velocity or almost equal nystagmus velocity on both sides during supine roll always led to an inconsistent diagnosis of horizontal canal BPPV (*N* = 15).	
Using only eye movements for diagnosing BPPV was never sufficient to consistently diagnose subjective BPPV during video reviews (*N* = 2).	Feedback of the patients was lacking during Video Reviews 1 and 2.
The presence of purely vertical nystagmus (downbeat or upbeat (*N* = 31) could lead to an inconsistent diagnosis (*N* = 11).	

* This analysis separately examined the left and right Dix–Hallpike test results to account for the characteristic nystagmus on each side.

**Table 5:** Additional recommendations for BPPV telemedicine protocol based on descriptive analysis (Table 4) and clinician’s reflection.

1. Record spontaneous nystagmus in both sitting and supine positions. 2. Record eye movement tracings to capture direction and velocity of the nystagmus. 3. Perform additional tests, such as the bow and lean test, to support the diagnosis of horizontal canal BPPV. 4. Report patient feedback during maneuvers.

## Data Availability

The data presented in this study are available on request from the corresponding author. The data are not publicly available due to privacy or ethical restrictions.

## Abbreviations

The following abbreviations are used in this manuscript:

BPPV	benign paroxysmal positional vertigo
SPV	slow-phase velocity
